# Planning Accuracy and Stem Offset Assessment in Digital Two-Dimensional Versus Three-Dimensional Planning in Cementless Hip Arthroplasty: A Systematic Review and Meta-Analysis

**DOI:** 10.3390/jcm13216566

**Published:** 2024-10-31

**Authors:** Francesco Rosario Parisi, Biagio Zampogna, Andrea Zampoli, Augusto Ferrini, Giorgio Albimonti, Alessandro Del Monaco, Pierangelo Za, Giuseppe Francesco Papalia, Rocco Papalia

**Affiliations:** 1 Operative Research Unit of Orthopaedic and Trauma Surgery, Fondazione Policlinico Universitario Campus Bio-Medico, Via Alvaro del Portillo 200, 00128 Rome, Italy; f.parisi@unicampus.it (F.R.P.); b.zampogna@policlinicocampus.it (B.Z.); andrea.zampoli@unicampus.it (A.Z.); augusto.ferrini@policlinicocampus.it (A.F.); giorgio.albimonti@unicampus.it (G.A.); alessandro.delmonaco@unicampus.it (A.D.M.); p.za@unicampus.it (P.Z.); r.papalia@policlinicocampus.it (R.P.); 2 Research Unit of Orthopaedic and Trauma Surgery, Department of Medicine and Surgery, Università Campus Bio-Medico Di Roma, Via Alvaro del Portillo 21, 00128 Rome, Italy; 3 BIOMORF Department of Biomedical, Dental, Morphological and Functional Images, University of Messina. A.O.U. Policlinico “G.Martino”, Via Consolare Valeria, 98124 Messina, Italy

**Keywords:** noninvasive surgery, prosthetic, preoperative planning

## Abstract

**Background/Objectives:** Total hip arthroplasty (THA) planning is crucial for restoring hip function and minimizing complications. The present systematic review and meta-analysis aimed to assess and compare the accuracy of 2D versus 3D preoperative planning in THA. **Methods**: The inclusion criteria were randomized controlled trials (RCTs) and observational studies (ROSs) published in English comparing the accuracy of 2D and 3D preoperative planning for total hip arthroplasty. We excluded review articles, registers, studies not written in English, studies that did not report the cup sizing accuracy or stem sizing accuracy or give a description of the preoperative planning method used, and non-comparative studies. In June 2024, following the PRISMA 2020 statement, a systematic review and a meta-analysis of the literature were conducted in PubMed, Scopus, and the Cochrane Library. The statistical analysis software Review Manager (RevMan) version 5.4 was used to perform the meta-analysis to compare the accuracy of 2D and 3D planning, and to assess the risk of bias, the ROBINS-I tool was used. **Results**: The analysis included 777 patients from six studies. The analysis showed that 3D planning offers superior precision compared to 2D planning, both for the cup (96.92% vs. 87.14%) and the stem (94.72% vs. 86.28%). The forest plots assessed a better trend for 3D planning in terms of exact size prediction and accuracy within ±1 size. **Conclusions**: The three-dimensional method was more precise and accurate than two-dimensional planning, both for the stem and the cup. It offered a detailed three-dimensional view of the patient’s anatomy. The main limitation was the challenge in finding homogeneous data regarding biomechanical parameters, surgical approaches, and different planning systems for both three-dimensional and two-dimensional methods.

## 1. Introduction

Total hip arthroplasty (THA) is recognized as a very successful treatment for osteoarthritis in its advanced stages, with the potential to significantly raise patient satisfaction and improve the functional results and their quality of life [1,2,3]. Total hip arthroplasty aims to restore normal hip anatomy, biomechanics, and function by replacing the femoral head, the acetabulum, and bearing surfaces [4,5,6]. A critical premise for hip arthroplasty surgery is performing a comprehensive evaluation in the decision-making process. When discussing preoperative planning in THA, it is wrong to think we are referring to the simple choice of the stem and cup size [7]. Planning is a complex process that starts from the patient’s first visit and continues with a careful and systematic radiographic analysis of the patient [8,9]. Of primary importance is the study of all anatomical–biomechanical parameters and the choice of the best-performing intervention strategy for the patient, helping to predict the position, size, and biomechanical parameters, in particular the femoral offset, and any operative difficulties [10,11]. Another primary goal in planning for hip arthroplasty surgery is restoring hip function while minimizing complications [12,13,14]. The long-term success of THA depends on decreasing the probability of problems such as dislocations, fractures, and limb length differences [15,16]. Accurate preoperative planning is essential for efficient THA, minimizing complications and technical and implant errors. Historically, preoperative planning has been based on two-dimensional (2D) templates, which estimated the implant size and location using traditional or digital radiographs [17,18,19]. The modern software program uses bidimensional pelvic X-rays with markers for digital planning [20,21,22]. It offers an option for appropriate stem and cup sizing for a patient’s anatomy [10]. Several studies have determined that digital planning is required before surgery [23]. According to the above and the vast literature on digital hip arthroplasty planning, numerous biomechanical characteristics must be considered in the decision-making process. These include limb length discrepancies, femoral offset, acetabular offset, and hip rotation center, which should be examined during preoperative planning [11,24,25,26]. Two-dimensional (2D) radiographic imaging is the most widely used support in digital planning today due to both its low cost and ease of use and the vast literature supporting its reliability and reproducibility [18,23,27]. Two-dimensional (2D) digital planning is performed on two-dimensional radiographs, with disadvantages in evaluating pathology and three-dimensional biomechanical parameters. In today’s world, advanced imaging techniques like computed tomography (CT) [28] and methods using two radiographs (the EOS system) [28,29,30] are revolutionizing the planning process for total hip arthroplasty. These techniques help us overcome the limitations of simple radiographs and show highly accurate and reproducible data for patients [31]. Three-dimensional (3D) preoperative planning with computed tomography (CT) scans has undoubted advantages over traditional planning based on two-dimensional radiographs [32,33,34]. Furthermore, this method reduces mistakes and inaccurate magnification [35,36]. Three-dimensional templating has several limits; some of the most important limitations are the increased radiation exposure and costs. Numerous studies are still needed to determine whether 3D planning enhances the long-term results compared to traditional 2D templating [37]. This review and meta-analysis aim to compare the accuracy and precision of 2D and 3D preoperative planning in total hip arthroplasty (THA).

## 2. Materials and Methods

**Figure 1:**
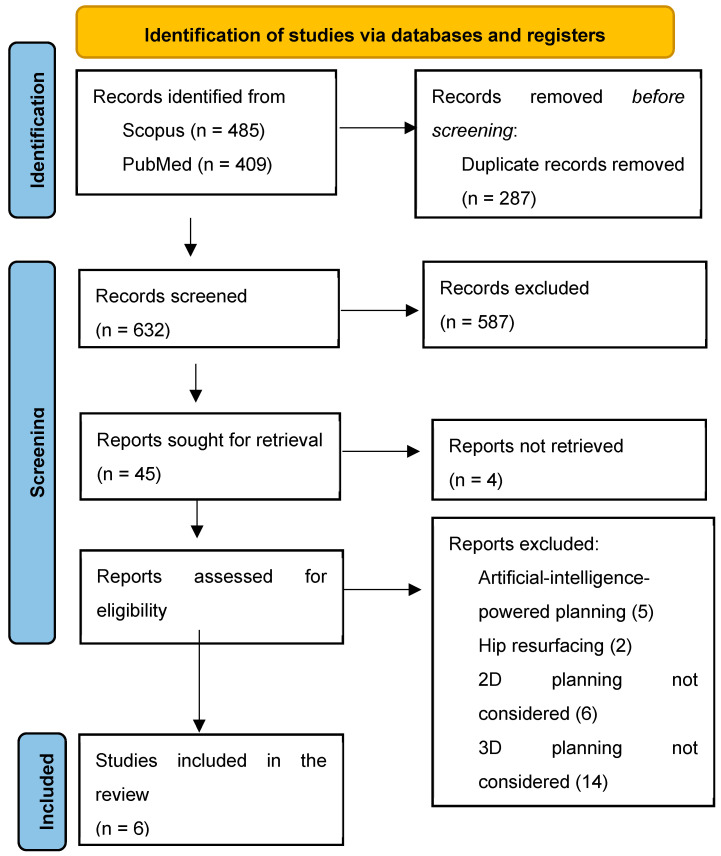
Preferred Reporting Items for Systematic Reviews and Meta-Analyses (PRISMA) flowchart.

This study was conducted following the methodology described in the 2020 guidelines of the Preferred Reporting Items for Systematic Reviews and Meta-Analyses (PRISMA) [38] (Figure 1).

### 2.1. Eligibility Criteria

The studies included were randomized controlled trials (RCTs) and retrospective or prospective studies written in English comparing 2D digital planning with 3D digital planning. Specifically, studies had to compare the accuracy of stem sizing, cup sizing, and stem offset prediction between the two planning modalities. Review articles, registers, and studies not written in English were excluded. Additionally, studies that did not report at least one of the following were excluded—accuracy of the cup sizing, accuracy of the stem sizing, or a description of how the preoperative planning was performed—as were non-comparative studies of the two methodologies. We also excluded studies that did not compare the radiological outcomes of 2D and 3D planning. To be included, studies had to clearly compare 2D and 3D planning techniques regarding their accuracy for both the stem and the cup.

### 2.2. The Literature Search

This systematic review and meta-analysis collected data from studies focused on adult patients undergoing total hip arthroplasty with preoperative planning in 2D or 3D. Two independent reviewers (F.R.P. and A.Z.) performed a literature search on July 30, 2024. The following databases were comprehensively searched: PubMed, Scopus, and Cochrane Library. Restrictions were applied to the search, considering only articles from 2000 to 2024. A third reviewer (G.F.P.) oversaw the research process. Titles and abstracts extracted using the previously reported search string were independently reviewed. Subsequently, the full manuscript of studies that met the inclusion criteria was examined, and eligible studies were definitively included.

### 2.3. Data Extraction and Collection

Two independent reviewers (F.R.P. and A.Z.) extracted the following data: the authors, type of study, year of publication, level of evidence (LOE), and the number of patients, with their gender, mean age, and body mass index. Regarding planning data, implant model and brand, stem and cup planning, and the planning software used were the information collected. In particular, implant planning accuracy for the cup and stem and accuracy of the planning for the lateral femoral offset were collected for both groups.

### 2.4. Statistical Analysis

Review Manager (RevMan) software version 5.4 was used to conduct the meta-analysis. Among the outcomes reported in all the comparative studies, cup and stem accuracy were assessed as dichotomous outcomes with their odds ratios (ORs) and 95% confidence intervals (CIs) using a random-effect model. The planning analysis was defined as exact if the planned and implanted implants were the same size. The accuracy was evaluated as the number of same-size implanted cups and stems or those ±1 size out of the total. All continuous variables were analyzed using means ± standard deviation or medians or interquartile ranges. The statistical significance of the results was fixed at *p* < 0.05.

### 2.5. Assessment of the Methodological Quality

Two independent reviewers (F.R.P. and G.F.P.) used the ROBINS-I tool to assess the risk of bias for the observational studies included [39].

## 3. Results

### 3.1. Results of the Search

The literature research identified 919 articles. After the removal of duplicates, 632 articles were screened for their titles and abstracts. The full text of 41 articles was read, and 35 were excluded for different reasons: artificial-intelligence-powered planning (n = 5); hip resurfacing (n = 2); 2D planning not being considered (n = 6); 3D planning not being considered (n = 14); and different outcomes or the accuracy not being specified (n = 8). Ultimately, six articles were included in this review (Figure 1). 

### 3.2. Included Studies

We included six studies: one randomized controlled trial (RCT), two randomized prospective comparative randomized studies, and three retrospective observational studies (ROSs). These studies compared the implanted size accuracy and the stem offset accuracy between two groups of patients who underwent total hip arthroplasty according to two-dimensional (2D) and three-dimensional (3D) preoperative planning. All the studies analyzed occurred between 2018 and 2024.

### 3.3. Demographic Data

The study included 777 patients (52.97% male and 47.03% female). The mean age of the patients was 63.01 ± 3.82 years. The BMI of the patients was 25.5 ± 2.56 Kg/m^2^ (Table 1).

### 3.4. Implant Used

An analysis of the papers included revealed that several models of cups and stems were used. Two studies used an Allofit cup (Zimmer) combined with a Zweymüller or Avenir (Zimmer) stem; the other associations were Optymis (Mathys), Fitmore (Zimmer), the SPP II anatomical stem (LINK), and the MIA stem (Smith & Nephew). Two studies used a Trident II (Stryker) cup combined with an Accolade II stem (Stryker). One study used a Dynacup (Corin) combined with a Meije stem (Corin). One study used a Delta cup (Ceramtec) combined with quadrangular and modular stems, depending on the planning technique used (2D or 3D).

### 3.5. Planning Software

Several planning software were investigated during the analysis. Two studies used the two-dimensional TraumaCad (Brainlab, Chicago, IL, USA) software and MediCad (Altdorf, Germany) software. Imagika (ViewTeck, Saint Maur, France) was used in only one case, in another case the analysis software used was not specified. Concerning three-dimensional digital planning, Mako planning software (Stryker Corp., Mako Surgical Corp., Ft. Lauderdale, FL, USA) was used in two studies, and HipEOS (Alphatec Holdings, Inc., Carlsbad, CA, USA) planning software was used in one study. The other three studies used 3D OPSInsight (Corin, Cirencester, UK), HipPlan (Symbios, Yverdon, Switzerland) and 3D ZedHip (Lexi, Japan) (Table 1).

### 3.6. Cup Accuracy

**Figure 2:**
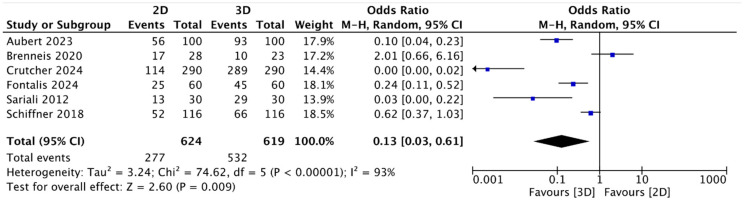
Forest plot comparing exact cup size prediction using three-dimensional (3D) and two-dimensional (2D) planning methods [40,41,42,43,44,45].

**Figure 3:**
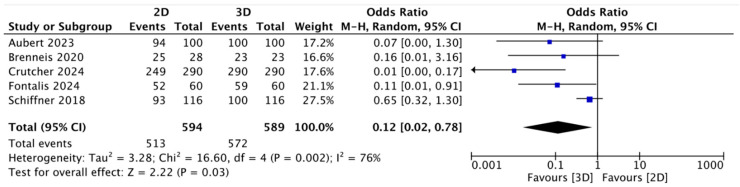
Forest plot comparing accuracy (range ± 1) of cup size prediction using three-dimensional (3D) and two-dimensional (2D) planning methods [40,41,42,43,45].

In the studies examined, the two-dimensional (2D) method planned the exact size (the same size implanted) in 47.53% of cases; concerning the mean accuracy (the same size implanted ± 1 size) of the 2D cup planning, this was 87.14% ± 5.06. The mean accuracy in the three-dimensional (3D) digital cup planning was 77.35%, and the accuracy (same size implanted ± 1 size) of 3D cup planning was 96.92% ± 6.03 (Table 1). The meta-analysis showed that for both the exact analysis group and the accuracy (range ± 1 size) group, higher-accuracy preoperative planning occurred with three-dimensional (3D) planning, with statistical significance (OR = 0.13; 95% CI: 0.03 to 0.61; *p* = 0.00001; I2 = 93) (Figure 2) (OR = 0.12; 95% CI: 0.02 to 0.78; *p* = 0.03; I2 = 76) (Figure 3).

### 3.7. Stem Accuracy

**Figure 4:**
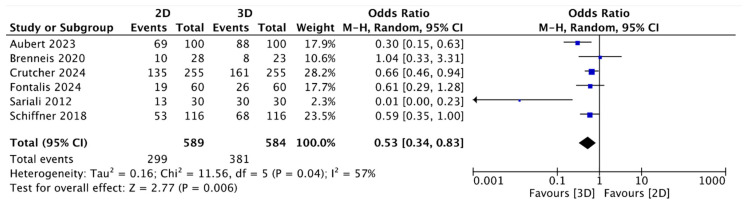
Forest plot comparing exact stem size prediction using three-dimensional (3D) and two-dimensional (2D) planning methods [40,41,42,43,44,45].

**Figure 5:**
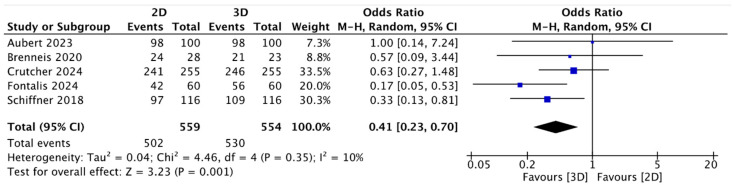
Forest plot comparing accuracy (range ± 1) of stem size prediction using three-dimensional (3D) and two-dimensional (2D) planning methods [40,41,42,43,45].

In the present analysis, the two-dimensional (2D) method planned the exact size (the same size of stem was implanted) with a mean of 46.33%; concerning the mean accuracy (same size implanted ± 1) for 2D stem planning, this was 86.28% ± 11.07. The mean accuracy for three-dimensional (3D) digital stem planning was 64.65%, and the accuracy (same size implanted ± 1) of 3D stem planning was 94.72% ± 2.71. The meta-analysis, as in the case of the acetabular cups, and the analysis of the accuracy of the stems showed data in favor of 3D planning (Table 1). The meta-analysis of the results for the stem showed higher accuracy in three-dimensional (3D) preoperative planning in terms of both the exact analysis (OR = 0.53, 95% CI: 0.34 to 0.83; *p* = 0.04; I2 = 57) (Figure 4) and the accuracy (range ± 1) (OR = 0.41, 95% CI: 0.23 to 0.70; *p* = 0.001; I2 = 10) (Figure 5), with statistical significance.

### 3.8. Stem Offset Prediction

**Figure 6:**
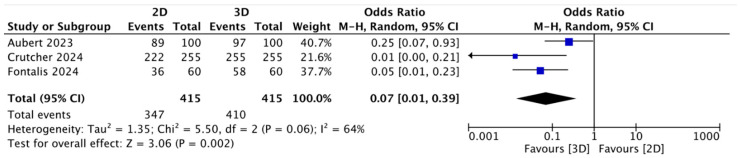
Forest plot comparing accuracy of stem offset prediction using three-dimensional (3D) and two-dimensional (2D) planning methods [41,42,43].

In the present study, we evaluated stem offset predictions with two-dimensional (2D) and three-dimensional (3D) planning methods. The mean value in stem offset planning was 77.1% using the 2D method and 97.77% with the 3D method (Table 1). The meta-analysis, as previously noted, showed higher accuracy in preoperative stem offset planning in the three-dimensional (3D) group, with statistical significance (OR = 0.07; 95% CI: 0.01 to 0.39; *p* = 0.002; I2 = 64) (Figure 6). 

### 3.9. Quality Assessment

All studies were assessed using the ROBINS-I tool (Table 2). Four studies were evaluated as having a low risk of bias, and two studies had a moderate risk of bias (Supplementary Materials).

## 4. Discussion

### 4.1. Overview

This systematic review and meta-analysis found that three-dimensional digital planning is a valuable method in the planning process for hip arthroplasty surgery. The group analyzed for which the three-dimensional method was employed to plan total hip arthroplasty demonstrated increased precision in both the exactness of the sizing and accuracy in the range of one size compared to those for which two-dimensional digital planning was used. In detail, this literature review and meta-analysis analyzed six comparative studies of the two methods, highlighting how the procedure based on three-dimensional imaging was found to be more accurate than that based on two-dimensional imaging in terms of both femoral stem and cup planning and restoring one of the most critical biomechanical parameters of the hip, the femoral offset.

### 4.2. Advantages of 3D Preoperative Planning

Three-dimensional digital planning has emerged as a method that guarantees essential advantages due to its intrinsic potential compared to the traditional and more widespread two-dimensional digital planning method [32]. These results overall are in line with the results of the most recent systematic reviews and meta-analyses, such as that of Habeeb Bishi et al. [46], where the accuracy of the two planning methods was evaluated considering both comparative and non-comparative studies and digital and analog two-dimensional planning methods were examined. All of this makes the preoperative decision-making process easier by minimizing errors and the effect of external variables that commonly affect the reliability of two-dimensional digital methods, such as poorly positioned landmarks, surgeons’ experience in planning, spinopelvic alignment, and altered magnification [35,47,48]. 

CT is widely used for three-dimensional planning and post-operative evaluations, providing high accuracy in analyzing versions of femoral and acetabular components [49]. The planning literature is vast and substantial [46,50,51,52,53]. In recent years, advanced imaging-based planning methods such as CT have become increasingly important [54,55]. The debate regarding its large-scale application is a topic of discussion at every meeting, thanks to surgical applications such as robots, which base their strength precisely on three-dimensional planning. The results of the current systematic review and meta-analysis are important in describing the relationship between the two most used methods in this panorama. It represents the first meta-analysis that has analyzed comparative studies.

### 4.3. Analysis of the Study

Analyzing the data from the meta-analysis (Figure 2, Figure 3, Figure 4 and Figure 5) shows how the data have evident statistical significance and favor three-dimensional digital planning in terms of all the factors analyzed. The only exception found, which did not change the final result, was the study by Brenneis et al. [40]. They reported that cup planning tended to be more precise with the two-dimensional method, even though the overall accuracy was better with the three-dimensional methodology. These results can partly be explained by the fact that it is the only one of the six studies analyzed that used the HipEOS planning system based on two low-dose coplanar radiographs for three-dimensional planning. The literature supporting its effectiveness is vast and almost comparable to that for other three-dimensional methods, such as CT, as demonstrated by the studies by Buller et al. [56], Knafo et al. [57], and Anderson et al. [58]. However, it represents a more modern method that has not been widely developed yet or applied at a large scale. The study by Brenneis et al. [40]. is also the only one that assessed two subcategories of stems: short and straight. They showed that three-dimensional digital planning is more accurate than two-dimensional digital planning when planning a short stem. This result can be explained considering that three-dimensional imaging methods give more detailed anatomical information on the metaphyseal region, the main anchoring region for a short stem [59]. Another interesting study considered in this meta-analysis was that of Fontalis et al. [41], which reported results in favor of three-dimensional digital planning in terms of exact and accurate planning in the range of one stem size and cup. Different parameters were studied in addition to the accuracy of the planning of the various components, such as the length of the legs, the position on the vertical and horizontal axes of the center of rotation (COR), and the global offset of the implant compared to the contralateral one. However, no statistically significant differences were found.

A sensitivity analysis evaluating the effect of different patterns of arthrosis, whether medial or postero-superior, on the positioning of the acetabular cup was of particular interest. The analysis of the subgroup of patients with medial arthrosis precisely defined how the three-dimensional planning allowed the COR to be less medialized and a global offset more like the contralateral one to be achieved. On the other hand, patients with superior and lateral OA had the worst translation of the COR and an altered global offset. These findings represent a tool for understanding and guiding two-dimensional planning. Analyzing the data from James P. Crutcher et al. [42], Aubert et al. [43], and Fontalis et al. [41], this meta-analysis showed that three-dimensional planning is a more precise method for choosing the offset of the stem.

Examining this parameter, all three studies highlighted how planning of the femoral stem neck offset using three-dimensional imaging was statistically significantly more accurate than using the two-dimensional method. The evidence found in this systematic literature review and meta-analysis also aligns with the results of studies that have only considered the three-dimensional planning method [32,46], showing the enormous technical advantage of this method compared to the previous generation.

### 4.4. Challenges and Cost–Benefit Considerations

In clinical practice, concerning the superiority and use of these innovations, there is always a point of conflict on topics such as the cost–benefit ratio of these methods and the technical and ethical difficulties. If the superiority of these applications is increasingly evident, it is also true that weak points, such as the high level of radiation and the non-homogeneity of the existing protocols, are still present [37,55,60,61,62]. Interesting and exemplifying are the findings of the working group of Angelika Ramush et al. [63], who showed how there are currently 17 CT scanning protocols, each with their own field of analysis of the pelvis and consequently differing amounts of radiation absorbed by the patient and each with their own definition of their anatomical reference parameters, which do not make the planning process homogeneous. As mentioned above, in addition to the problems related to radiation, despite low-emission protocols, the debate regarding the cost–benefit ratio of these methods based on advanced imaging is not of secondary importance [64,65]. Over the years, cost–benefit analyses have highlighted how these plans, although they are advantageous from a technical point of view, do not justify the high costs they require in their application, especially in centers with low operating volumes. In this scenario, a new method, the EOS system, was presented based on two X-rays. A system with evident low use of radiation allowed for an exact three-dimensional analysis of the patient’s anatomy [28,56,57]. 

### 4.5. Future Directions and Limitations

Although it is easy to apply and involves low radiation emissions, this method is still high-cost today due to its recent introduction and therefore narrow distribution. Despite the improved accuracy demonstrated by the literature and its results, such as the present meta-analysis and the ever-increasing application of robotic methods, three-dimensional planning is not yet supported by concrete evidence that suggests its significant clinical advantage. Artificial intelligence and large-scale data analysis applications could help in these debated issues [66]. There is increasing scientific and academic evidence regarding the ability of both machine learning and artificial intelligence systems to support the preoperative planning decision process [67,68]. These new technologies may allow us to apply two-dimensional imaging methods better in the future and, at the same time, help us find solutions in robotic surgery and three-dimensional imaging that overcome the current problems with their large-scale applicability. There are several limitations to this study. One of its strengths, which is its analysis of comparative studies only, is also a limitation due to the challenge in finding homogeneous data. Only six comparative studies were included. Another limitation is the limited analysis of all the biomechanical parameters considered during planning and the inclusion of different planning systems, both three-dimensional and two-dimensional. Furthermore, another limitation is that planning and surgery were performed by different surgeons, and the surgical approaches were not the same in all the studies, and in some studies, they were not specified. These observations are of extreme importance, particularly regarding 2D planning, which appears to be more subject to inter-individual oscillations than 3D planning.

## 5. Conclusions

The present meta-analysis highlights the results of three-dimensional planning in THA. The three-dimensional technique was more precise and accurate, and it allowed for a complete analysis and view of the patient’s anatomy, overcoming the limits of the two-dimensional method. Despite its technical benefits, debates persist about its costs–benefits and the technical and ethical difficulties of its large-scale implementation. To justify the widespread adoption of three-dimensional planning in primary hip arthroplasties, further research is essential to demonstrate the clinical advantages resulting from its greater precision concretely.

## Figures and Tables

**Table 1:** Patient characteristics, demographic data, planning software used, and stem and cup size planning accuracy in the 3D and 2D groups.

Author	Year	Counry	(n)	Cup (n)	Stem (n)	Comparison	Number	Software	Age		BMI (Kg/m^2^)		Implanted Accuracy Cup %			Implanted Accuracy Stem %			Cup Accuracy	Stem Accuracy	Accuracy Offset
									Mean	sd	Mean	sd	Same	±1	≥2	Same	±1	≥2	Same ± 1	Same ± 1	
Brenneis et al. [40]	2020	Germany	51	51	51	2D Planning	28	TraumaCAD	63.5		27.7		60.7	28.6	10.7	35.7	50	14.3	89.3	85.7	
						3D Planning	23	HipEOS	60.2		27.8		43.5	56.5	0	34.8	56.5	8.7	100	91.3	
Fontalis et al. [41]	2024	UK	60	60	60	2D Planning	60	TraumaCAD	65		26.7		41.7	45	13.3	31.7	38.3	30	86.7	69.6	60
						3D Planning	60	Mako (Stryker)					75	23.4	1.6	43.4	50	6.6	98.4	93.4	96.3
Crutcher et al. [42]	2024	USA	290	290	255	2D Planning	290	-					39	46.5	14.5	52.9	41.6	5.5	85.5	94.5	82.3
						3D Planning	290	Mako (Stryker)					99.7	0.3	0	63.1	33.3	3.6	100	96.9	100
Aubert et al. [43]	2023	France	200	200	200	2D Planning	100	MediCad	66		21.8		56	38	6	69	29	2	94	98	89
						3D Planning	100	3D OPSInsight	63		22.1		93	7	0	88	10	2	100	98	97
Sariali et al. [44]	2012	France	60	60	60	2D Planning	30	Imagika	57.2	13	25.8	6.7	43			43					
						3D Planning	30	HipPlan	60	15	27.1	3.7	96			100					
Schiffner et al. [45]	2018	Germany	116	116	116	2D Planning	116	MediCad	69.2				44.8	35.4	19.8	45.7	37.9	16.3	80.2	83.6	
						3D Planning	116	3D ZedHip					56.9	29.3	13.8	58.6	35.4	6	86.2	94	

**Table 2:** Risk of bias assessment evaluated with ROBINS-I.

STUDY	RISK OF BIAS (ROBINS-I)
BRENNEIS (2020) [40]	Low
FONTALIS (2024) [41]	Low
CRUTCHER (2024) [42]	Low
AUBERT (2023) [43]	Moderate
SARIALI (2012) [44]	Low
SCHIFFNER (2018) [45]	Moderate

## Supplementary Materials

The following supporting information can be downloaded at: https://www.mdpi.com/article/10.3390/jcm13216566/s1, Table S1: Robins I quality assessment.

## References

[1] Snell D.L., Dunn J.A., Hooper G. (2024). Associations between pain, function and quality of life after total hip arthroplasty. Int. J. Orthop. Trauma Nurs..

[2] Learmonth I.D., Young C., Rorabeck C. (2007). The operation of the century: Total hip replacement. Lancet.

[3] Zampogna B., Papalia G.F., Parisi F.R., Luciano C., Gregori P., Vorini F., Marinozzi A., Farsetti P., Papalia R. (2023). Early return to activity of daily living after total hip arthroplasty: A systematic review and meta-analysis. Hip Int. J. Clin. Exp. Res. Hip Pathol. Ther..

[4] Mellon S.J., Liddle A.D., Pandit H. (2013). Hip replacement: Landmark surgery in modern medical history. Maturitas.

[5] Petis S., Howard J.L., Lanting B.L., Vasarhelyi E.M. (2015). Surgical approach in primary total hip arthroplasty: Anatomy, technique and clinical outcomes. Can. J. Surg. J. Can. Chir..

[6] Müller M.E. (1992). Lessons of 30 years of total hip arthroplasty. Clin. Orthop..

[7] Colombi A., Schena D., Castelli C.C. (2019). Total hip arthroplasty planning. EFORT Open Rev..

[8] Conn K.S., Clarke M.T., Hallett J.P. (2002). A simple guide to determine the magnification of radiographs and to improve the accuracy of preoperative templating. J. Bone Jt. Surg Br..

[9] Scheerlinck T. (2010). Primary hip arthroplasty templating on standard radiographs. A stepwise approach. Acta Orthop. Belg..

[10] Di Martino A., Rossomando V., Brunello M., D’Agostino C., Pederiva D., Frugiuele J., Pilla F., Faldini C. (2023). How to perform correct templating in total hip replacement. Musculoskelet. Surg..

[11] Lecerf G., Fessy M.H., Philippot R., Massin P., Giraud F., Flecher X., Girard J., Mertl P., Marchetti E., Stindel E. (2009). Femoral offset: Anatomical concept, definition, assessment, implications for preoperative templating and hip arthroplasty. Orthop. Traumatol. Surg. Res. OTSR.

[12] Vigdorchik J.M., Sharma A.K., Jerabek S.A., Mayman D.J., Sculco P.K. (2021). Templating for Total Hip Arthroplasty in the Modern Age. J. Am. Acad. Orthop. Surg..

[13] Haddad F.S., Masri B.A., Garbuz D.S., Duncan C.P. (1999). The Prevention of Periprosthetic Fractures in Total Hip and Knee Arthroplasty. Orthop. Clin. N. Am..

[14] Knight J.L., Atwater R.D. (1992). Preoperative planning for total hip arthroplasty. Quantitating its utility and precision. J. Arthroplast..

[15] Shah N., Hodgkinson J. (2014). Why do Orthopaedic surgeons get sued after total hip replacement?. Bone Jt..

[16] Jang S.J., Vigdorchik J.M., Windsor E.W., Schwarzkopf R., Mayman D.J., Sculco P.K. (2022). Abnormal spinopelvic mobility as a risk factor for acetabular placement error in total hip arthroplasty using optical computer-assisted surgical navigation system. Bone Jt. Open.

[17] Eggli S., Pisan M., Müller M.E. (1998). The value of preoperative planning for total hip arthroplasty. J. Bone Jt. Surg. Br..

[18] Dammerer D., Keiler A., Herrnegger S., Putzer D., Strasser S., Liebensteiner M. (2021). Accuracy of digital templating of uncemented total hip arthroplasty at a certified arthroplasty center: A retrospective comparative study. Arch. Orthop. Trauma Surg..

[19] Iorio R., Siegel J., Specht L.M., Tilzey J.F., Hartman A., Healy W.L. (2009). A Comparison of Acetate vs. Digital Templating for Preoperative Planning of Total Hip Arthroplasty. J. Arthroplast..

[20] Lindgren J.U., Rysavy J. (1992). Restoration of femoral offset during hip replacement. A radiographic cadaver study. Acta Orthop. Scand..

[21] Sershon R.A., Diaz A., Bohl D.D., Levine B.R. (2017). Effect of Body Mass Index on Digital Templating for Total Hip Arthroplasty. J. Arthroplast..

[22] Luger M., Hochgatterer R., Klotz M.C., Hipmair G., Gotterbarm T., Schauer B. (2022). Digital templating for the implantation of a curved short hip stem with an anterolateral MIS approach shows gender differences in digital templating. Arch. Orthop. Trauma Surg..

[23] Smith J.B.V., Bishi H., Wang C., Asopa V., Field R.E., Sochart D.H. (2021). The accuracy and reliability of preoperative digital 2D templating in prosthesis size prediction in uncemented versus cemented total hip arthroplasty: A systematic review and meta-analysis. EFORT Open Rev..

[24] LaCour M., Ta M., Nachtrab J., Nguyen T., Komistek R. (2024). Determination of optimal component positioning in THA using 3D preoperative planning. J. Orthop. Res. Off. Publ. Orthop. Res. Soc..

[25] Bachour F., Marchetti E., Bocquet D., Vasseur L., Migaud H., Girard J. (2010). Radiographic preoperative templating of extra-offset cemented THA implants: How reliable is it and how does it affect survival?. Orthop. Traumatol. Surg. Res. OTSR.

[26] Flecher X., Ollivier M., Argenson J.N. (2016). Lower limb length and offset in total hip arthroplasty. Orthop. Traumatol. Surg. Res..

[27] Della Valle A.G., Padgett D.E., Salvati E.A. (2005). Preoperative planning for primary total hip arthroplasty. J. Am. Acad. Orthop. Surg..

[28] Huang J., Zhu Y., Ma W., Zhang Z., Shi W., Lin J. (2020). A Novel Method for Accurate Preoperative Templating for Total Hip Arthroplasty Using a Biplanar Digital Radiographic (EOS) System. JBJS Open Access.

[29] Mayr H.O., Schmidt J.P., Haasters F., Bernstein A., Schmal H., Prall W.C. (2021). Anteversion Angle Measurement in Suspected Torsional Malalignment of the Femur in 3-Dimensional EOS vs. Computed Tomography—A Validation Study. J. Arthroplast..

[30] Rivière C., Lazic S., Dagneaux L., Van Der Straeten C., Cobb J., Muirhead-Allwood S. (2018). Spine–hip relations in patients with hip osteoarthritis. EFORT Open Rev..

[31] Lattanzi R., Baruffaldi F., Zannoni C., Viceconti M. (2004). Specialised CT scan protocols for 3-D pre-operative planning of total hip replacement. Med. Eng. Phys..

[32] Moralidou M., Di Laura A., Henckel J., Hothi H., Hart A.J. (2020). Three-dimensional pre-operative planning of primary hip arthroplasty: A systematic literature review. EFORT Open Rev..

[33] Ghotra S.S., Cottier Y., Bruguier C., Dominguez A., Monnin P., dos Reis C.S. (2024). A Pilot Study to Identify Suitable MRI Protocols for Preoperative Planning of Total Hip Arthroplasty. Eur. J. Radiol..

[34] Crone T.P., Cornelissen B.M.W., Van Oldenrijk J., Bos P.K., Veltman E.S. (2024). Intraoperative application of three-dimensional printed guides in total hip arthroplasty: A systematic review. World J. Orthop..

[35] Olmedo-Garcia N.I., Vergara J.L.M., Miralles T.L.A., Andrés J.V.S., Vives A.M., Renovell E.C., Beltran V.G. (2018). Assessment of magnification of digital radiographs in total HIP arthroplasty. J. Orthop..

[36] Holliday M., Steward A. (2021). Pre-operative templating for total hip arthroplasty: How does radiographic technique and calibration marker placement affect image magnification?. J. Med. Radiat. Sci..

[37] Huppertz A., Radmer S., Asbach P., Juran R., Schwenke C., Diederichs G., Hamm B., Sparmann M. (2011). Computed tomography for preoperative planning in minimal-invasive total hip arthroplasty: Radiation exposure and cost analysis. Eur. J. Radiol..

[38] Page M.J., McKenzie J.E., Bossuyt P.M., Boutron I., Hoffmann T.C., Mulrow C.D., Shamseer L., Tetzlaff J.M., Akl E.A., Brennan S.E. (2021). The PRISMA 2020 statement: An updated guideline for reporting systematic reviews. Syst. Rev..

[39] Sterne J.A., Hernán M.A., Reeves B.C., Savović J., Berkman N.D., Viswanathan M., Henry D., Altman D.G., Ansari M.T., Boutron I. (2016). ROBINS-I: A tool for assessing risk of bias in non-randomised studies of interventions. BMJ.

[40] Brenneis M., Braun S., van Drongelen S., Fey B., Tarhan T., Stief F., Meurer A. (2021). Accuracy of Preoperative Templating in Total Hip Arthroplasty With Special Focus on Stem Morphology: A Randomized Comparison Between Common Digital and Three-Dimensional Planning Using Biplanar Radiographs. J. Arthroplast..

[41] Fontalis A., Yasen A.T., Kayani B., Luo T.D., Mancino F., Magan A., Plastow R., Haddad F.S. (2024). Two-Dimensional Versus Three-Dimensional Preoperative Planning in Total Hip Arthroplasty. J. Arthroplast..

[42] Crutcher J.P., Hameed D., Dubin J., Mont M.A. (2024). Comparison of three-versus two-dimensional pre-operative planning for total hip arthroplasty. J. Orthop..

[43] Aubert T., Galanzino G., Gerard P., Le Strat V., Rigoulot G., Lhotellier L. (2023). Accuracy of Preoperative 3D vs. 2D Digital Templating for Cementless Total Hip Arthroplasty Using a Direct Anterior Approach. Arthroplast. Today.

[44] Sariali E., Mauprivez R., Khiami F., Pascal-Mousselard H., Catonné Y. (2012). Accuracy of the preoperative planning for cementless total hip arthroplasty. A randomised comparison between three-dimensional computerised planning and conventional templating. Orthop. Traumatol. Surg. Res..

[45] Schiffner E., Latz D., Jungbluth P., Grassmann J.P., Tanner S., Karbowski A., Windolf J., Schneppendahl J. (2019). Is computerised 3D templating more accurate than 2D templating to predict size of components in primary total hip arthroplasty?. HIP Int..

[46] Bishi H., Smith J.B.V., Asopa V., Field R.E., Wang C., Sochart D.H. (2022). Comparison of the accuracy of 2D and 3D templating methods for planning primary total hip replacement: A systematic review and meta-analysis. EFORT Open Rev..

[47] Grammatopoulos G., Innmann M., Phan P., Bodner R., Meermans G. (2023). Spinopelvic challenges in primary total hip arthroplasty. EFORT Open Rev..

[48] Schapira B., Madanipour S., Iranpour F., Subramanian P. (2023). Accuracy of Total Hip Arthroplasty Templating Using Set Calibration Magnifications. Cureus.

[49] Fujishiro T., Hayashi S., Kanzaki N., Hashimoto S., Kurosaka M., Kanno T., Masuda T. (2014). Computed tomographic measurement of acetabular and femoral component version in total hip arthroplasty. Int. Orthop..

[50] Holzer L.A., Scholler G., Wagner S., Friesenbichler J., Maurer-Ertl W., Leithner A. (2019). The accuracy of digital templating in uncemented total hip arthroplasty. Arch. Orthop. Trauma Surg..

[51] Zampogna B., Parisi F.R., Zampoli A., Prezioso A., Vorini F., Laudisio A., Papalia M., Papapietro N., Falez F., Papalia R. (2024). Accuracy of two-dimensional digital planning in uncemented primary hip arthroplasty: Monocentric analysis of eight hundred implants. Int. Orthop..

[52] Shaarani S.R., McHugh G., Collins D.A. (2013). Accuracy of digital preoperative templating in 100 consecutive uncemented total hip arthroplasties: A single surgeon series. J. Arthroplast..

[53] Gamble P., de Beer J., Petruccelli D., Winemaker M. (2010). The Accuracy of Digital Templating in Uncemented Total Hip Arthroplasty. J. Arthroplast..

[54] Mainard D., Barbier O., Knafo Y., Belleville R., Mainard-Simard L., Gross J.B. (2017). Accuracy and reproducibility of preoperative three-dimensional planning for total hip arthroplasty using biplanar low-dose radiographs: A pilot study. Orthop. Traumatol. Surg. Res..

[55] Hassani H., Cherix S., Ek E.T., Rüdiger H.A. (2014). Comparisons of Preoperative Three-Dimensional Planning and Surgical Reconstruction in Primary Cementless Total Hip Arthroplasty. J. Arthroplast..

[56] Buller L.T., McLawhorn A.S., Maratt J.D., Carroll K.M., Mayman D.J. (2021). EOS Imaging is Accurate and Reproducible for Preoperative Total Hip Arthroplasty Templating. J. Arthroplast..

[57] Knafo Y., Houfani F., Zaharia B., Egrise F., Clerc-Urmès I., Mainard D. (2019). Value of 3D Preoperative Planning for Primary Total Hip Arthroplasty Based on Biplanar Weightbearing Radiographs. BioMed Res. Int..

[58] Anderson C.G., Brilliant Z.R., Jang S.J., Sokrab R., Mayman D.J., Vigdorchik J.M., Sculco P.K., Jerabek S.A. (2022). Validating the use of 3D biplanar radiography versus CT when measuring femoral anteversion after total hip arthroplasty: A comparative study. Bone Jt. J..

[59] Klim S.M., Reinbacher P., Smolle M.A., Hecker A., Maier M., Friesenbichler J., Leithner A., Leitner L., Draschl A., Lewis J. (2023). Femoral Anteversion in Total Hip Arthroplasty: Retrospective Comparison of Short- and Straight-Stem Models Using CT Scans. J. Clin. Med..

[60] Huppertz A., Lembcke A., Sariali E.H., Durmus T., Schwenke C., Hamm B., Sparmann M., Baur A.D. (2015). Low Dose Computed Tomography for 3D Planning of Total Hip Arthroplasty: Evaluation of Radiation Exposure and Image Quality. J. Comput. Assist. Tomogr..

[61] Kaiser D., Hoch A., Rahm S., Stern C., Sutter R., Zingg P.O. (2023). Combining the advantages of 3-D and 2-D templating of total hip arthroplasty using a new tin-filtered ultra-low-dose CT of the hip with comparable radiation dose to conventional radiographs. Arch. Orthop. Trauma Surg..

[62] Geijer M., Rundgren G., Weber L., Flivik G. (2017). Effective dose in low-dose CT compared with radiography for templating of total hip arthroplasty. Acta Radiol..

[63] Ramesh A., Di Laura A., Henckel J., Hart A. (2023). The variability of CT scan protocols for total hip arthroplasty: A call for harmonisation. EFORT Open Rev..

[64] Zhang Z., Luo Y., Zhang J., Zhang C., Wang X., Chen J., Chai W. (2024). Can Robotic Arm-assisted Total Knee Arthroplasty Remain Cost-effective in Volume-based Procurement System in China? A Markov Model-based Study. Orthop. Surg..

[65] Rajan P.V., Khlopas A., Klika A., Molloy R., Krebs V., Piuzzi N.S. (2022). The Cost-Effectiveness of Robotic-Assisted Versus Manual Total Knee Arthroplasty: A Markov Model–Based Evaluation. JAAOS J. Am. Acad. Orthop. Surg..

[66] Huo J., Huang G., Han D., Wang X., Bu Y., Chen Y., Cai D., Zhao C. (2021). Value of 3D preoperative planning for primary total hip arthroplasty based on artificial intelligence technology. J. Orthop. Surg..

[67] Zampogna B., Torre G., Zampoli A., Parisi F., Ferrini A., Shanmugasundaram S., Franceschetti E., Papalia R. (2024). Can machine learning predict the accuracy of preoperative planning for total hip arthroplasty, basing on patient-related factors? An explorative investigation on Supervised machine learning classification models. J. Clin. Orthop. Trauma.

[68] Xie H., Yi J., Huang Y., Guo R., Liu Y., Kong X., Chai W. (2024). Application and evaluation of artificial intelligence 3D preoperative planning software in developmental dysplasia of the hip. J. Orthop. Surg..

