# Supplementary material for: Planning Accuracy and Stem Offset Assessment in Digital Two-Dimensional Versus Three-Dimensional Planning in Cementless Hip Arthroplasty: A Systematic Review and Meta-Analysis
Source: J Clin Med. 2024 Oct 31;13(21):6566. doi: 10.3390/jcm13216566 (PMC11546058; doi:10.3390/jcm13216566)
Supplement: Supplementary file 1 [file jcm-13-06566-s001.zip › jcm-3281052-supplementary.pdf]

	DOMAIN 1	DOMAIN 2	DOMAIN 3	DOMAIN 4	DOMAIN 5	DOMAIN 6	DOMAIN 7	Overall risk of bias
	Bias due to confounding	Bias in selection of participants into the study	Bias in classification of interventions	Bias due to deviations from intended interventions	Bias due to missing data	Bias in measurement of outcomes	Bias in selection of the reported result	
Brenneis (2020)	1.1 N LOW	2.1 N LOW<br>2.2 Y LOW	3.1 Y LOW<br>3.2 Y LOW<br>3.3 N LOW	4.1 N LOW	5.1 Y LOW<br>5.2 N LOW<br>5.3 N LOW	6.1 N LOW<br>6.2 N LOW<br>6.3 Y LOW<br>6.4 N LOW	7.1 N LOW<br>7.2 N LOW<br>7.3 N LOW	**LOW**
Fontalis (2024)	1.1 N LOW	2.1 N LOW<br>2.2 Y LOW	3.1 Y LOW<br>3.2 Y LOW<br>3.3 N LOW	4.1 N LOW	5.1 Y LOW<br>5.2 N LOW<br>5.3 N LOW	6.1 N LOW<br>6.2 N LOW<br>6.3 Y LOW<br>6.4 N LOW	7.1 N LOW<br>7.2 N LOW<br>7.3 N LOW	**LOW**
Crutcher (2024)	1.1 N LOW	2.1 N LOW<br>2.2 Y LOW	3.1 Y LOW<br>3.2 Y LOW<br>3.3 N LOW	4.1 N LOW	5.1 Y LOW<br>5.2 N LOW<br>5.3 N LOW	6.1 N LOW<br>6.2 N LOW<br>6.3 Y LOW<br>6.4 N LOW	7.1 N LOW<br>7.2 N LOW<br>7.3 N LOW	**LOW**
Aubert (2023)	1.1 N LOW	2.1 N LOW<br>2.2 Y LOW	3.1 Y LOW<br>3.2 Y LOW<br>3.3 N LOW	4.1 N LOW	5.1 Y LOW<br>5.2 N LOW<br>5.3 N LOW	6.1 PY HIGH<br>6.2 Y HIGH<br>6.3 Y LOW<br>6.4 N LOW	7.1 N LOW<br>7.2 N LOW<br>7.3 N LOW	**MODERATE**
Sariali (2012)	1.1 N LOW	2.1 N LOW<br>2.2 Y LOW	3.1 Y LOW<br>3.2 Y LOW<br>3.3 N LOW	4.1 N LOW	5.1 Y LOW<br>5.2 N LOW<br>5.3 N LOW	6.1 N LOW<br>6.2 N LOW<br>6.3 Y LOW<br>6.4 N LOW	7.1 N LOW<br>7.2 N LOW<br>7.3 N LOW	**LOW**
Schiffner (2018)	1.1 N LOW	2.1 N LOW<br>2.2 Y LOW	3.1 Y LOW<br>3.2 Y LOW<br>3.3 N LOW	4.1 N LOW	5.1 Y LOW<br>5.2 N LOW<br>5.3 N LOW	6.1 PY HIGH<br>6.2 Y HIGH<br>6.3 Y LOW<br>6.4 N LOW	7.1 N LOW<br>7.2 N LOW<br>7.3 N LOW	**MODERATE**
